# Emerging roles for ROS and RNS – versatile molecules in plants

**DOI:** 10.1093/jxb/erx236

**Published:** 2017-08-10

**Authors:** Ismail Turkan

**Affiliations:** Ege University, Faculty of Science, Department of Biology, Izmir, BO, Turkey

**Keywords:** Abiotic stress, biotic stress, defence, development, free radicals, great oxidation event, oxygen toxicity, reactive nitrogen species (RNS), reactive oxygen species (ROS)

**Increased cellular levels of potentially harmful reactive oxygen species (ROS) and reactive nitrogen species (RNS) come about as plants contend with harsh environmental conditions. At low concentrations, however, ROS and RNS can act as signals for the regulation of growth and development and for defence against biotic and abiotic stress. Indeed, we now know that they are involved in nearly every facet of plant metabolism and cell function. This virtual issue introduces active research in this rapidly moving field, as well as discussing the gaps in our knowledge and the technical developments which are opening up new vistas.**

Atmospheric oxygen levels increased with the rise of oxygen-evolving photosynthesis on Earth, which resulted in the great oxidation event 2.4 billion years ago (Inupakutika *et al.*, 2016). This increase resulted in two events involving life on the planet: first, the evolution of physiological mechanisms to utilize oxygen to produce energy (oxidative phosphorylation) and, second, the development of defensive mechanisms against oxygen toxicity. During the evolution of higher plants from their ancient unicellular ancestors, plants found solutions of increasing complexity to cope with reactive oxygen species (ROS), toxic by-products of oxidative metabolism, and adapt their metabolism to this selective pressure (see Box 1). The evolution of ROS metabolism, which preceded the great oxidation event and originated as early as 4.1–3.6 billion years ago, has recently been reviewed by Inupakutika *et al.* (2016).

**Box 1. Sites/mechanisms of ROS production in the cell and known interactions**

ROS are by-products of plant metabolism even under normal conditions, and various types can be produced in different sub-cellular compartments. Mechanisms of ROS production are common to all plants, although mechanisms for counteracting them or perceiving them as signals differ. Although toxic at high concentrations, they can act as signals for the regulation of growth and development and for defence against biotic and abiotic stress at low concentrations. Types and amounts of ROS vary from organelle to organelle due to wide distribution of metabolic processes. Only H_2_O_2_ can diffuse across membranes; signalling by O_2_^.−^ and ^1^O_2_ requires some sort of intermediate, generally a breakdown product of an organic molecule that is oxidized by these types of ROS.

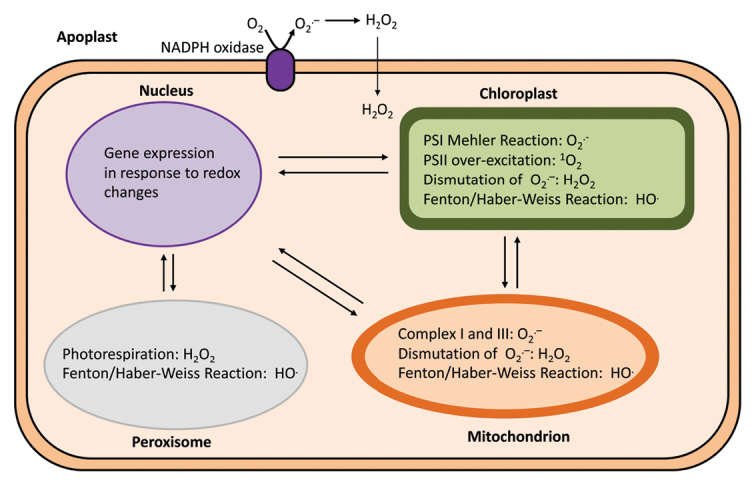

Free radicals are defined as chemical species which contain one or more unpaired electrons. The term, ROS, describes all pro-oxidants which originate from O_2_, but there are also other reactive molecules that can originate from other elements, such as nitrogen (hence reactive nitrogen species, RNS). In plant cells, ROS and/or RNS can be formed through several reactions in different compartments, for example during photosynthesis in chloroplasts, cellular respiration in mitochondria, photorespiration in peroxisomes, and during different oxidation–reduction reactions in the cytosol.

The presence of free radicals in living systems was shown in the 1950s and 1960s. At the time they were suggested to be responsible for cell deterioration and senescence due to their damaging effects on lipids, proteins and nucleic acids (Commoner *et al.*, 1954; McCord and Fridovich, 1969). Since then, the study of oxidative stress physiology has been a key research theme in plant and animal biology, as well as medicine (Fridovich, 1978). In the 1990s, researchers realized that ROS might also act as signalling molecules, in spite of their damaging effects, and this hypothesis shaped the progress of ongoing research in the area. Development of microarrays in the 2000s enabled the demonstration of dramatic changes in the transcriptome of plants in response to low levels of ROS, strengthening this idea. It is now well known that ROS and RNS act as mediators of different signalling pathways; they are involved in growth and development, reproduction, nutrient sensing, and defensive responses against abiotic and biotic factors. A major part of ROS signal transduction relies on redox regulation of thiols on proteins. Therefore, proteins that can regulate thiols or use them as reducing power – such as thioredoxins, peroxiredoxins, glutaredoxins and sulfiredoxins – have been the subject of much research, reviewed by Waszczak *et al.* (2015) and Sevilla *et al.* (2015).

Excellent reviews by del Rio (2015) and Mignolet-Spruyt *et al.* (2016) discuss the chemistry of ROS and RNS with special emphasis on their site-specific production. In addition, del Rio (2015) presents a detailed chronology of ROS and RNS research, summarizing how we got to where we are now.

## Abiotic stress

The role of ROS and RNS in response to environmental stresses has been studied for nearly three decades, and there are a large number of published papers on this area. Following the trends in the development of ROS research, work on abiotic stress initially focused on the damaging effects of these molecules under stress conditions and associated counteracting mechanisms. However, later on, it was demonstrated that signalling roles of ROS and RNS are vital for induction of defence responses. Although there is a vast literature on this topic, molecular mechanisms involving ROS and RNS during abiotic and biotic stresses are still unclear.

Drought and salinity are two major environmental problems that limit crop yield, and mitigating the effects of these stresses is already an important issue with the accelerating effects of climate change. To understand the upstream regulatory events in response to drought, researchers target members of different families of transcription factors, and in most of these cases stress-related transcription factors are found to be related to ROS metabolism. Two examples show the roles of stress-related transcription for regulation of ROS metabolism. First, Zhang *et al.* (2015) demonstrated that overexpressing the ABRE-binding factor (PtABF) in trifoliate orange (*Poncirus trifoliate*) results in increased dehydration tolerance. PtABF was shown to interact with ICE1 (Inducer of CBF Expression 1) and affect stomatal development. In addition, antioxidant enzymes and polyamine biosynthesis were induced in PtABF-overexpressing plants, causing less cellular damage under dehydration. In another study, Fang *et al.* (2015) similarly demonstrated that overexpression of SNAC3 results in tolerance to high temperature, drought and oxidative stress tolerance in rice. Moreover, they showed that SNAC3 can bind directly to the promoters of *CATA*, *APX8* and *RbohF* genes, which are important components of ROS defence and signalling.

In the case of salt stress, the role of ROS and RNS in regulating membrane transporters, counteracting the ionic effects of salinity, is an interesting and emerging area. For example, Jayakannan *et al.* (2015) showed that, under salt stress, NPR1-dependent salicylic acid (SA) signalling controls Na entry into roots and long-distance transport into the shoot by regulating ROS-activated NSCC channels. The lack of NPR1-dependent SA signalling in the *npr1-5* mutant showed sensitivity to ROS treatments, and results showed that NPR1 is an important factor that mediates salt-induced H_2_O_2_ production in plants. Moreover, when the salinity stress tolerance of three *Brassica* species (*B. napus, B. juncea*, *B. oleracea*) was evaluated, reduced sensitivity of *B. napus* root K^+^-permeable channels to ROS was found to be one of the dominant factors that make this species salt tolerant when compared to the other two (Chakraborty *et al.*, 2016). Accordingly, Shabala (2017) presents a Viewpoint article covering interaction between ROS and membrane transporters; this raises questions about K^+^-dependent signalling, which might be triggered by Ca^2+^ and ROS. There are also gaps in our knowledge of the mechanisms which induce defensive mechanisms, such as the production of high levels of SODs. In their paper, Xing *et al.* (2015) report on the elucidation of a mechanism that is responsible for induction of the Arabidopsis *FSD2* and *FSD3* genes, which encode the FeSOD enzyme. They demonstrate that expression of these two genes is influenced by MEKK1 via MKK5–MPK6-coupled signalling. Recent research also elucidated new functions of NADPH oxidases related to high atmospheric CO_2_-dependent alleviation of salt stress. Yi *et al.* (2015) demonstrated that silencing the tomato *RBOH1* gene abolished high CO_2_-induced salt tolerance and increased transpiration rates, as well as enhancing Na^+^ accumulation in the plants. These results provide evidence of the necessity for apoplastic H_2_O_2_ under elevated-CO_2_ conditions for regulating stomatal aperture.

As with drought and salinity, heat stress is a widely observed phenomenon under natural conditions and ROS and RNS are again involved in many mechanisms underlying high temperature responses and tolerance. Recently, Cheng *et al.* (2016) showed that 2-Cys PRXs are involved in heat stress responses by regulating autophagosome formation and ascorbate-glutathione metabolism in chloroplasts, which is particularly novel in terms of the signalling role of 2-Cys PRX in autophagy. Moreover, Qiao *et al.* (2015) showed that OsANN1, an annexin protein with calcium-binding and ATPase activities, regulates H_2_O_2_ levels in rice under heat stress. OsANN1 also interacts with OsCDPK24, a calcium-dependent protein kinase, which might provide an additional layer of regulation during heat stress.

Exposure to high light changes the redox balance of chloroplasts and causes the production of a variety of ROS, including ^1^O_2_ and O_2_^.−^, and H_2_O_2_ or HO^.^. These ROS cause different transcriptomic profiles in the plants and have various signalling roles. In his excellent review, Dietz (2015) gives a timeline of events during six hours of high light stress in terms of redox states, levels of reactive oxygen species, metabolites, and hormones and gene expression. Also, in their detailed work, Borisova-Mubarakshina *et al.* (2015) revealed a new signalling role for H_2_O_2_ and found that hydrogen peroxide contributes to triggering adaptive responses of the photosynthetic apparatus, which include reduction of the antenna size of photosystem II.

## Biotic stress

Under natural conditions, plants are not usually exposed to just one stress, such as high temperature or drought, but a combination. Similarly, abiotic stresses are generally accompanied by biotic factors such as bacteria, fungi or herbivores. Hence, cross-tolerance mechanisms to multiple stresses and their relationship with ROS metabolism are also a topic of interest. In their innovative review, Foyer *et al.* (2016) focus on cross-tolerance to multiple stresses and look at how the abiotic environment influences plant responses to attack by aphids; they conclude that these responses involve overlap and interaction points between hormone, ROS and RNS signalling pathways, and that they have features in common with abiotic stress responses.

Although the connection between the NADPH oxidase-mediated oxidative burst and pathogen resistance has been extensively studied, underlying regulative mechanisms for this burst have been unclear. In their elegant work, Morales *et al.* (2016) investigated the expression patterns of RbohD and RbohF, which mediate diverse physiological processes including pathogen resistance, and observed differential expression patterns throughout plant development and during the immune response. Moreover, promoter-swap experiments (between RbohD and RbohF) demonstrated that the promoter region of RbohD is required for ROS production in response to pathogens.

Accumulated knowledge in the literature puts chloroplasts in a central position as integrators of environmental signals and vital defence organelles where biosynthesis and transmission of pro-defence signals occur during plant immune responses. In their review, Serrano *et al.* (2016) highlight interorganellar communication as a crucial process for amplification of the immune response and its relationship with the chloroplastic ROS burst.

## Growth and development

Since life forms including plants evolved in the presence of ROS on Earth, it would be logical to assume that an intimate relationship exists between developmental processes and ROS. Influences of ROS and RNS on growth are usually integrated with the action of hormones such as auxin, brassinosteroids, gibberellins, abscisic acid, ethylene, strigolactones, salicylic acid, and jasmonic acid. In their review article, Xia *et al.* (2015) describe the crosstalk between ROS and hormonal signalling, with an emphasis on the central role of ROS production and accumulation in plant hormone-mediated signalling and action in response to developmental and environmental stimuli. Moreover, an insight is provided into the integration nodes that involve Ca^2+^-dependent processes and mitogen-activated protein kinase phosphorylation cascades. In addition to this, Xu *et al.* (2016) carried out experiments with creeping bentgrass involving isopentenyltransferase (ipt), the rate-limiting enzyme in cytokinin biosynthesis; they demonstrated that overexpression of ipt with a promoter activated under senescence facilitated cytokinin-enhanced ROS scavenging through antioxidant accumulation and induction of alternative respiration pathways, which resulted in enhanced root growth under drought stress. In a similar context, Sanz *et al.* (2015) provide a compilation of the interactions which have been described between NO and phytohormones during early plant development processes such as seed dormancy and germination, hypocotyl elongation and root development.

## Perspectives

We now know that ROS and RNS are involved in nearly every facet of plant metabolism and cell function, yet there is still a lack of fundamental knowledge as to how these reactive molecules act as such specific signals. Technical developments in top-down approaches such as RNA-seq and proteomics have paved the way for progress, especially for discovering new transcription factors and protein–protein interactions, but still we need spatial data about ROS and RNS production in the cell and new insights into their specific functions. Similarly, the development of new microscopy techniques and *in vivo* optical manipulation of intracellular structures is creating new opportunities for investigating organellar ROS and RNS production and their functions in the cell.
